# Toxicological Use of Hypodermic Syringe

**Published:** 1875-02

**Authors:** 


Toxicological Use of Hypodermic Syringe.—We have received the following note from Dr. John E. Walker, of Greenesboro, Georgia:

"An attempt at poisoning was detected in the following manner, viz: The water contained in the house pail was found to be bitter, and from circumstances was supposed to contain strychnine. The ordinary tests failing to discover it, a large quantity was injected under the skin of a dog, which produced all of the toxicological effects of strychnine. A second dog was injected with strychnine from a drug store, and like symptoms were produced. Thus an attempt to destroy an entire family was detected, and the perpetrator, a negro girl, who confessed the crime, was brought to deserved punishment. The experiments were conducted by myself in 1868. Such use of the hypodermic syringe I have not found any mention of in my reading."

